# Regional Disparities in Japan’s Home Healthcare Resources: A Retrospective Observational Study Using Nationwide Data from 2014 to 2020

**DOI:** 10.1007/s11606-024-09285-6

**Published:** 2025-01-07

**Authors:** Yu Sun, Nobuo Sakata, Masao Iwagami, Satoru Yoshie, Ryota Inokuchi, Jun Hamano, Nanako Tamiya

**Affiliations:** 1 Department of Health Services Research, Institute of Medicine, University of Tsukuba, Tsukuba, Japan; 2 Health Services Research and Development Center, University of Tsukuba, Tsukuba, Japan; 3 Department of Primary Care and Medical Education, Institute of Medicine, University of Tsukuba, Tsukuba, Japan; 4 Heisei Medical Welfare Group Research Institute, Tokyo, Japan; 5 Department of home healthcare, Setagaya Memorial Hospital, Tokyo, Japan; 6 Institute of Gerontology, The University of Tokyo, Tokyo, Japan; 7 Institute for Future Initiatives, The University of Tokyo, Tokyo, Japan; 8 Department of Health Policy and Management, School of Medicine, Keio University, Tokyo, Japan; 9 School of Medicine, Hiroshima University, Hiroshima, Japan; 10 Institute of Medicine, University ot Tsukuba, Tsukuba, Japan

**Keywords:** home healthcare services, regional disparities, policy

## INTRODUCTION

Population aging has caused a global shift from hospital-based to home-based care^1^ with Japan at the forefront due to its rapid societal aging. The Japanese government has promoted home healthcare through policies such as introducing home care support clinics and hospitals (HCSCs) in 2006 and enhanced HCSCs in 2012.^2^ Recently, we summarized the details of HCSCs.^2^ Briefly, home healthcare in Japan involves regular home visits by physicians covered by health insurance. All HCSCs must provide a 24-hour home visiting care system, while enhanced HCSCs are designed to deliver higher-quality care, particularly for emergency visits and end-of-life care, and receive higher fees than conventional HCSCs.^2^ Consequently, we found that compared to home healthcare with non-HCSCs, HCSCs (especially enhanced) provide more emergency house calls, reduce hospitalizations and re-hospitalizations, and enable expected deaths at home.^2,3^

However, whether these HCSC-related policies have been equally implemented across Japan is unknown. Regional disparities may exist, and HCSCs may be more prevalent in urban. Thus, the present study aimed to investigate regional disparities in home healthcare resources in Japan, and their temporal changes from 2014 to 2020.

## METHODS

We used publicly available data from the Ministry of Health, Labour, and Welfare for 2014 (first survey year after the introduction of enhanced HCSCs in 2012), 2017, and 2020 (most recent survey year), which includes information on medical institutions and older adults. The analysis unit was a “secondary medical area” as defined by the Japanese government and used to determine the medical provision system and resource distribution.^4^ The Japanese government sometimes reclassifies secondary medical areas; thus, the study included 325 areas (from 335 in 2020) not reclassified between 2014 and 2020. As home healthcare resource indicators, we estimated the numbers of (i) all medical institutions offering home healthcare, (ii) all HCSCs, and (iii) enhanced HCSCs per older adult population (i.e., number of residents ≥ 65 years) in each secondary medical area in 2014, 2017, and 2020. We used Lorenz curves and estimated Gini coefficients to assess regional disparities, a common method for analyzing geographic distribution inequalities. As an additional analysis, we classified 325 secondary medical areas as urban, middle, and rural (depopulated)^5^ and examined the distribution and temporal trends of home healthcare resources.

## RESULTS

**Figure 1:**
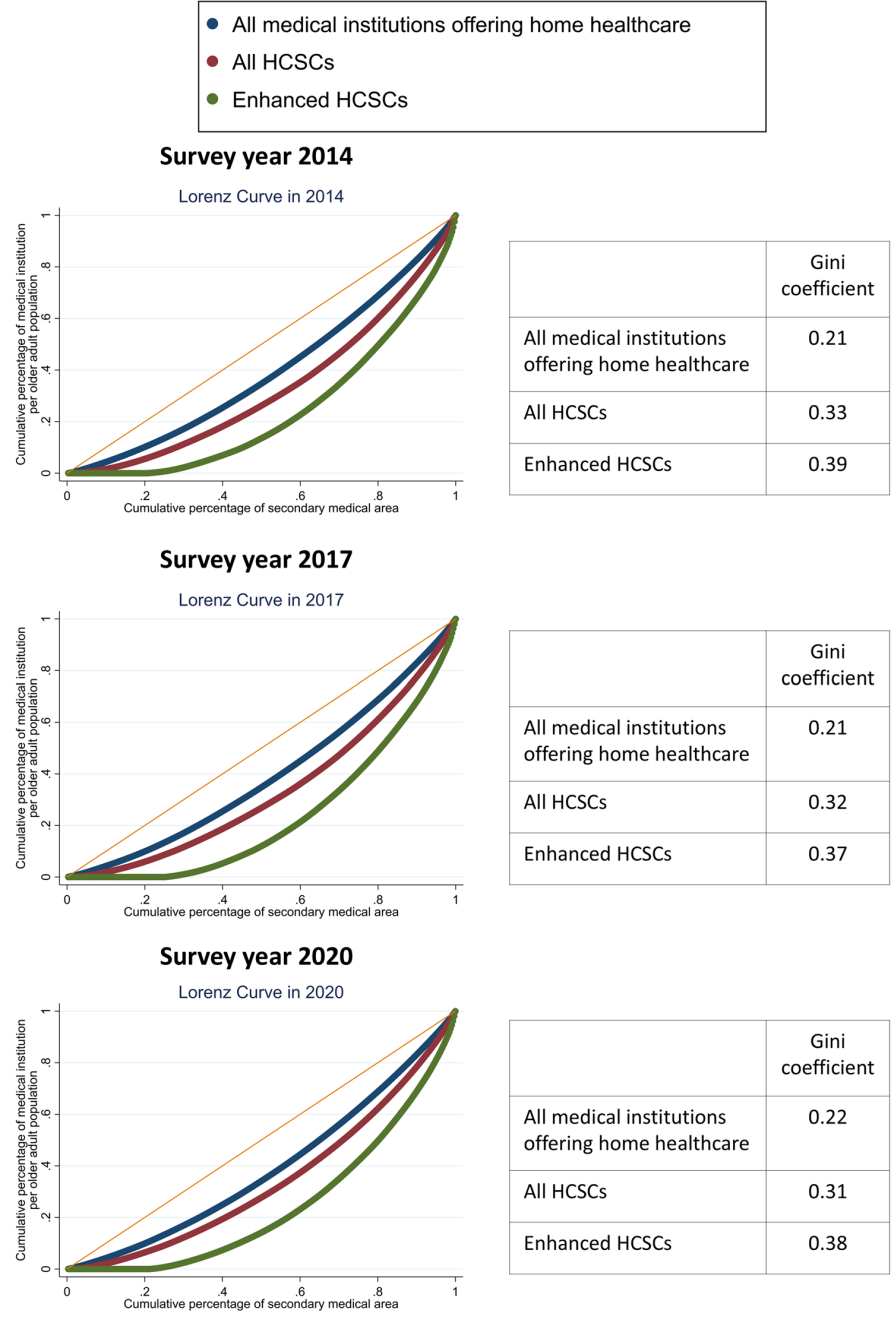
Lorenz curves and Gini coefficients for home healthcare resource indicators in 2014, 2017, and 2020. Abbreviations: HCSCs, home care support clinics and hospitals.

**Figure 2:**
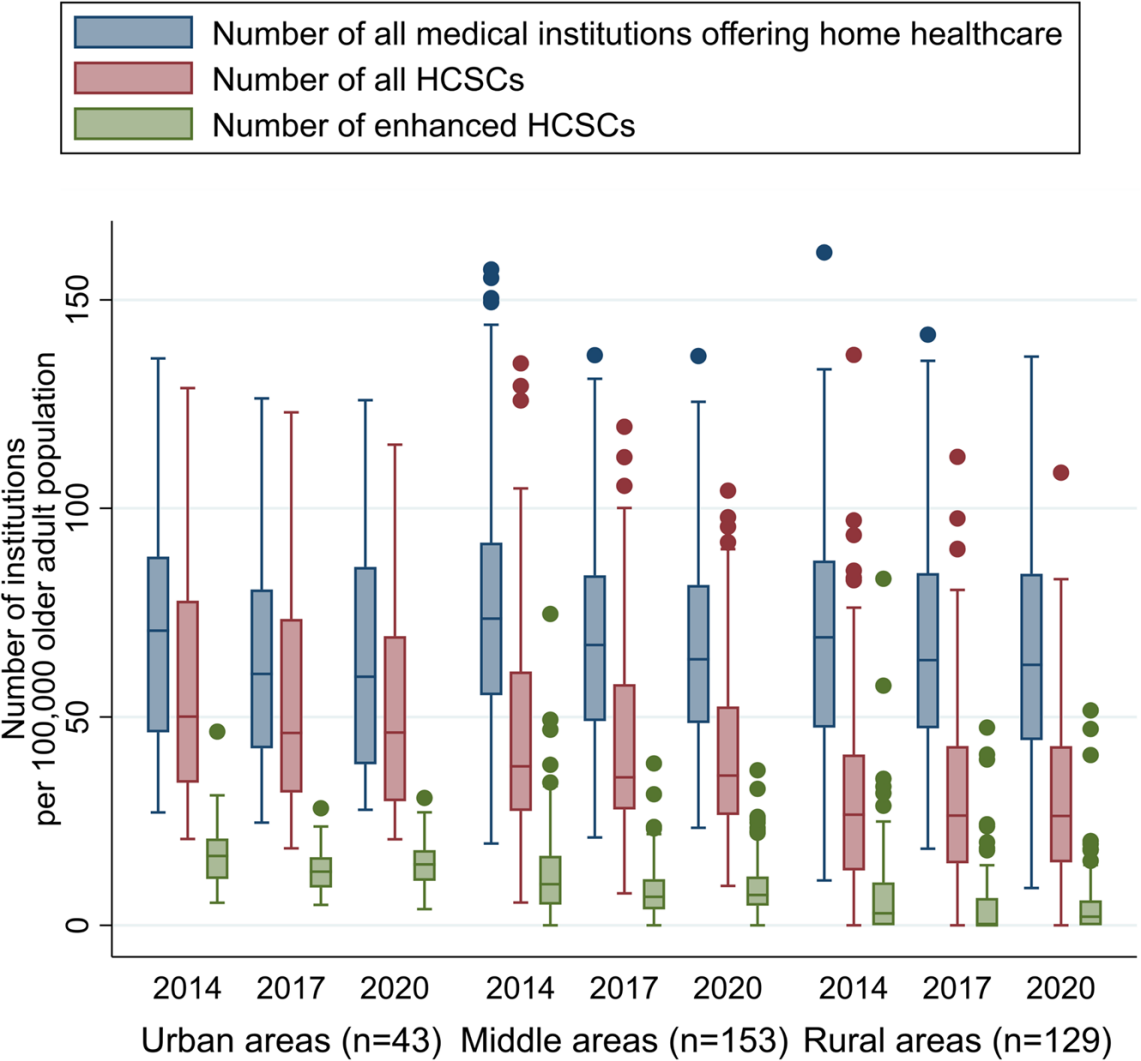
Graphical representation of the distribution and temporal trend of home healthcare resources among urban, middle, and rural (depopulated) areas. Abbreviations: HCSCs, home care support clinics and hospitals. Note: The urban group includes areas with a population of one million or more or a population density of 2000 people per square kilometer or more. The middle group included areas with a population of 200,000 or more or a population between 100,000 and 200,000, with a population density of 200 people per square kilometer or more. The rural (depopulated) group includes areas that do not fall into either category.

Figure 1 shows the Lorenz curve and estimated Gini coefficients, suggesting regional disparities for medical institutions offering home visits. The disparities were larger for HCSCs, particularly enhanced HCSCs. Moreover, regional disparities rarely changed between 2014 and 2020. Additional analyses indicate HCSCs (particularly enhanced) were less prevalent in rural than in urban areas similarly in 2014, 2017, and 2020 (Fig. 2).

## DISCUSSION

This study identified considerable regional disparities in home healthcare resources in Japan, with higher concentrations of (enhanced) HCSCs in urban areas compared to rural areas. Several factors likely contribute to this disparity. Urban areas have closer patient residences, making home visits more efficient, while rural areas’ scattered homes increase travel time and reduce profitability.^6^ Additionally, securing physicians and maintaining a 24-hour on-call system in rural areas can be challenging due to limited resources.^7^ Instead, general clinics may tend to conduct home visits to meet patient demand and support home healthcare in these areas. However, general clinics, primarily operated by solo-practice physicians, typically extend their outpatient services to include home healthcare.^6^ Therefore, they can visit only a limited number of patients and are often unable to manage complex medical requirements such as home oxygen therapy and palliative care for terminal cancer.^6^ Furthermore, these physicians face a heavy burden,^7^ which is a critical concern.

Several measures can help reduce these disparities. Implementing additional fees for enhanced HCSCs, based on population density, could incentivize home healthcare in rural areas. Promoting telemedicine systems could also reduce travel burdens for providers, improving the feasibility of rural home healthcare.

In conclusion, our findings highlight persistent regional disparities in home healthcare resources in Japan from 2014 to 2020, with significant HCSCs (especially enhanced) resource limitations in rural areas. Targeted measures are essential to address these disparities and support the aging population effectively.

## Data Availability

All data used in this study are publicly available. The data can be accessed from the following sources: • Regional data collection for home healthcare https://view.officeapps.live.com/op/view.aspx?src=https%3A%2F%2Fwww.mhlw.go.jp%2Fcontent%2F10800000%2F001316849.xlsx&wdOrigin=BROWSELINK • Population census in 2000 https://www.e-stat.go.jp/dbview?sid=0003445099=00200521&tstat=000001136464&collect_area=200&metadata=1&data=1

## Declarations:

### Disclaimer:

The funder played no role in the conception, design, implementation, or reporting of this study.
